# Transient regional osteoporosis of the ankle with shifting bone marrow edema pattern within the joint^☆^

**DOI:** 10.1016/j.radcr.2022.05.089

**Published:** 2022-06-17

**Authors:** Yasser Ragab, Yasser Emad, Sara Hassanein, Johannes J. Rasker

**Affiliations:** a Radiology Department, Faculty of Medicine, Cairo University, Kasr Al-Ainy St, 11562 Cairo, Egypt; b Rheumatology Department, Faculty of Medicine, Cairo University, Kasr Al-Ainy St, 11562 Cairo, Egypt.; c Diagnostic Radiology Department, Faculty of Medicine, Assiut University, Assuit, Egypt; d Faculty of Behavioral, Management and Social Sciences, Department Psychology, Health and Technology, University of Twente, Drienerloolaan 5, 7522NB Enschede, the Netherlands

**Keywords:** Transient regional osteoporosis, Ankle, Shifting bone marrow edema, Magnetic resonance imaging (MRI)

## Abstract

We describe a case of bone marrow edema (BME) shifting within one ankle joint in a 35-year-old - male patient. He presented with increasing pain and no history of trauma. Clinically no local swelling was found and laboratory findings and plain x-ray studies were normal. He did not improve on non-steroidal anti-inflammatory drugs for 2 weeks. A Gadolinium enhanced magnetic resonance imaging showed no evidence of synovitis, but BME was observed in the talus and transient regional osteoporosis was diagnosed. The patient was treated conservatively by protective partial weight bearing of the affected joint and he showed partial improvement after 6 months of daily treatment with Calcitonin Salmon nasal spray. A magnetic resonance imaging after 6 months showed that the BME had shifted anteriorly with complete resolution at the initial site. Transient regional osteoporosis is a rare self-limiting syndrome characterized by sudden onset of joint pain, functional limitations and spontaneous recovery, without preceding trauma. The condition may present as one episode affecting only one joint or recurrent episode that may affect multiple joints. BME between different compartments of the same joint can occur and has been reported only in a few case reports in the knee joint. The case is discussed and the literature is reviewed.

## Introduction

Bone marrow edema (BME) is a radiological term that describes a region that has low signal intensity on T1-weighted MRI but high signal intensity on T2 MRI or in short tau inversion recovery sequences [1,2]. BME affecting the hip joint(s) is neither a specific MRI finding nor a specific diagnosis [1]. BME can be found in both adult and pediatric populations, with various underlying etiologies such as inflammatory arthropathy, transient osteoporosis of the hip (TOH), avascular necrosis, acute stress fractures, primary bone neoplasms, myeloproliferative bone marrow disorders like leukemia and hemoglobinopathies [1,2].

TOH, also known as transient BME (TBME), is most common in middle-aged men and most commonly occurs after minor trauma or sports injuries, primarily affecting the hip joint. TOH tends to improve with complete resolution of the BME pattern following Alendronate therapy, which belongs to the class of drugs known as bisphosphonates [3,4].

Transient regional osteoporosis is a rare self-limiting syndrome characterized by sudden onset of joint pain, and functional limitations and spontaneous recovery. The condition may present as one episode affecting only one joint or recurrent episode that may affect multiple joints, described as transient regional migratory osteoporosis. BME between different compartments of the same joint can occur and has been reported only in a few case reports in the knee joint [5,6].

In this report we describe a case of shifting BME within an ankle joint.

## Case summary

**Fig. 1:**
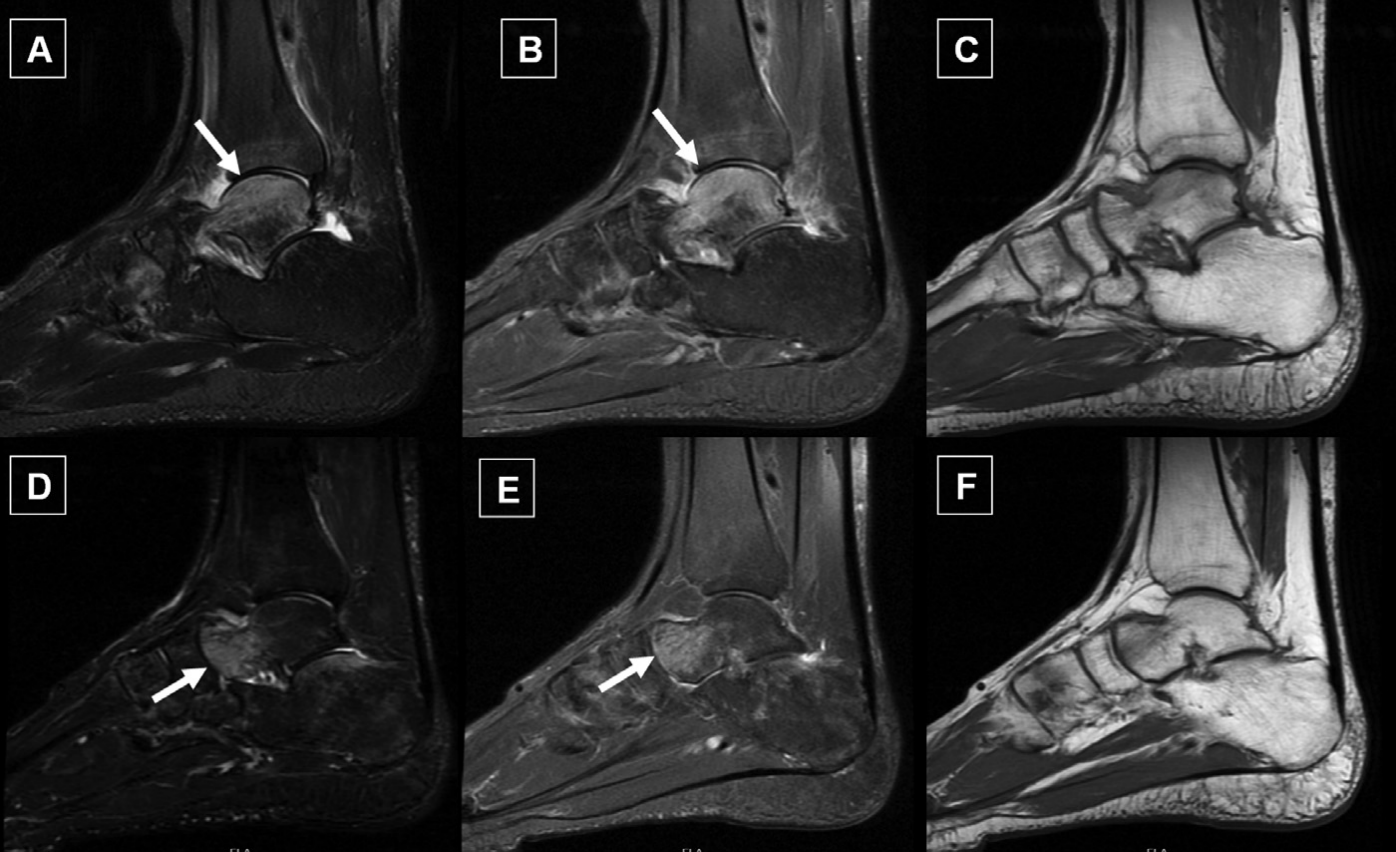
Sagittal MRI planes: (A) STIR, (B) PD, and (C) *T1* sequences of the ankle joint showing intense bone marrow edema (BME) in the dome of the talus bone (white arrows) and dark signal in *T1* (C); Sagittal MRI planes of the same ankle joint, (D) STIR, (E) PD, and (F) *T1* sequences, show that the BME shifted anteriorly in a follow-up MRI, after 6 months of treatment and partial clinical recovery (white arrow).

A 35-year-old male patient presented with an insidious onset of right ankle pain that gradually progressed over one month with no history of trauma or falling. There was no history of psoriasis and nor family history of rheumatological disorders. Clinically and after rheumatological examination, there was no evidence of ankle swelling suggestive of active synovitis, and there was no functional limitation of the affected joint. Other peripheral joints and the axial spine, particularly the sacroiliac joints showed no abnormalities. Initial laboratory investigations showed normal values of ESR first hour and normal CRP levels. Complete blood count was ordered to exclude blood dyscrasia and showed normal components values (hemoglobin, hematocrit, total and differential white blood cell count and normal platelets count). Rheumatoid factor, and anti-citrullinated protein (anti-CCP) antibodies were negative with normal serum calcium, and bone specific alkaline phosphatase levels and normal kidney function. Plain x-rays of the affected ankle in anteroposterior (AP), mortise, and lateral views, as well as x-rays of the left foot in AP, lateral, and oblique views, revealed normal radiographic features. After 2 weeks of short courses of nonsteroidal anti-inflammatory drugs, the patient's symptoms persisted, and Gadolinium enhanced magnetic resonance imaging (MRI) was ordered to rule out subclinical synovitis or other intra articular pathology. The first MRI study showed no evidence of synovial enhancement or joint effusion suggestive of occult synovitis, however, a BME pattern was observed in the dome of the talus bone (Figs. 1A–C). In view of the clinical and enhanced MRI findings we considered the diagnosis of transient regional osteoporosis of the ankle with BME pattern with no underlying etiology. The patient was treated conservatively by protective partial weight bearing of the affected joint and Calcitonin nasal spray on daily basis as nasal puffs (Miacalcic Nasal 200 IU, Novartis, Switzerland), together with active vitamin-D and calcium carbonate 500 mg daily. The patient showed partial improvement of his condition and a follow-up MRI study was carried out after 6 months to assess the response to therapy. Surprisingly the BME was shifted anteriorly with complete resolution of BME on the initial location in site the first MRI study (Figs. 1D–F). The medication was continued for half a year until the patient became totally pain-free.

## Discussion

The first use of the term “bone marrow edema” was by Wilson et al [7] in 1988,in patients with debilitating knee and hip pain, they described ill-defined bone marrow hyperintensities on T2-weighted MR images. The authors named this condition BME due to a "lack of a better term and to emphasize the condition's generic character."

BME syndromes (BMES) are a common cause of severe bone and joint pain. The disease is typically self-limiting, with symptoms resolving spontaneously over a 6- to 12-month period. BMES primarily affect the femoral head, but it can also occur in the knee, foot, or, less frequently, other locations. Patients report pain at rest and under stress, as well as a limited range of motion in the affected joint [8].

Recurrence of BME in other joints, usually on the contralateral side, may occur in some patients. These cases have been referred to as “regional migratory osteoporosis” or “regional migrating BMES”. Only a few case studies have described a shifting BME pattern within different compartments of the same knee joint [5,8,9].

BMES are often used to refer to transient clinical conditions with unknown pathogenic mechanisms, including TOH, regional migratory osteoporosis, and reflex sympathetic dystrophy. BMES primarily may affect the hip, knee, and ankle joints. Many hypotheses have been proposed to explain the disorder's pathogenesis, but the etiology of BMES remains unknown [10].

Concerning the hip joint BME pattern involving the hip is neither a specific MRI finding nor a specific diagnosis. BME affecting the hip can be encountered in a wide range of clinical situations [1]. BME of the femoral head and neck was recently found to be positively correlated with structural damage and synovitis in the cam type of femoro-acetabular impingement, but not in the pincer femoro-acetabular impingement type. [11]. Thromboembolism, obstruction of arteriolar inflow or venous outflow, altered lipid metabolism, and decreased fibrinolysis are some of the other commonly discussed theories [12,13].

BME intra-articular shift is a very rare disease that may lead clinicians to suspect an aggressive disease. Aigner et al [8] reviewed 8 patients (4 women and 4 men) with unilateral BMES in the knee. BME observed on MRI in all patients shifted within the same joint, from the medial to the lateral femoral condyle or the adjacent bone. After the initial detection of BME, 7 patients were given conservative therapy, including limited weight-bearing, for 3 weeks, while one patient underwent surgical core decompression twice. According to the authors, intra-articular shifting BMES is a very rare condition. Treatment with intravenous iloprost in combination with reduced weight-bearing appears also to accelerate the natural improvement of the disease in many cases [14]. In recent reports [3,4], treating TOH with an antiresorptive agent (alendronate) reduced the duration of the illness compared to the natural history of the disease, with complete resolution of the BME pattern on follow-up MRI. The use of alendronate to treat TOH was based on findings reported by McCarthy [15], who described biopsy specimens from 19 cases of transient regional osteoporosis at various joints. The presence of osteoclastic bone resorption, which was active in 14 of the 19 cases studied, was the most significant histopathological finding reported. Alendronate is chemically related to inorganic pyrophosphate; the latter inhibits osteoclastic bone resorption, whereas alendronate inhibits bone resorption specifically [16]. Alendronate efficacy in TOH is linked to inhibiting activated osteoclastic activity and thus preventing bone resorption, which can lead to femoral head collapse and eventually avascular necrosis. It is also has a strong analgesic effect on bone, which is still an important factor to consider in this domain [17].

In bone marrow cultures, calcitonin can inhibit osteoclast differentiation from precursor cells as well as the fusion of mononucleated precursors to form multinucleated cells [19]. Furthermore, it inhibits bone resorption by inducing contraction and inhibiting osteoclast motility (Q effect), which occurs within 1 minute, and is followed by a more gradual retraction of the osteoclasts (R effect) [20]. Calcitonin's therapeutic efficacy in our patient would be explained by the latter mechanisms of action, which would inhibit osteoclastic activity within the BME lesion, as previously documented by histopathological examination by McCarthy [15].

In our report, we describe a beneficial effect of calcitonin nasal spray which improved the pain in the affected ankle joint, yet the BME pattern resolved from the dome of the talus bone and shifted anteriorly as seen on follow-up MRI. The latter is rarely reported in the ankle joint. As in our patient, one patient was described as having migratory osteoporosis within the foot and ankle with a shifting BME pattern. The initial MRI revealed that BME had affected the cuboid and the base of the fifth metatarsal bone. A 12-month MRI study revealed that the BME from the cuboid and fifth metatarsal had resolved, but that a new BME had developed in the posterior calcaneus [21]. The latter findings are consistent with a shifting BME pattern, as evidenced by our patient's MRI findings.

In a previous report [18], modification of treatment with intramuscular calcitonin for 2 months led to an improvement of the knee pain in a case with transient migratory osteoporosis, and after 12 months of treatment follow-up MRI revealed reduction in the size of BME pattern initially detected. The authors described diffuse BME involving the external condyle in their case, and a few months later, shifting of the BME was detected in the ipsilateral internal condyle. In another MRI study, BME extended in the internal femoral condyle of the contralateral knee joint and later in the external condyle. The latter course usually corresponds to a shifting BME pattern as seen in our patient.

## Conclusion

Transient regional osteoporosis with shifting BME pattern has been described in a few cases in the knee joint, but rarely reported in the ankle joint, which may be due to a lack of follow-up MRI studies to document such a pattern within the same joint.

## Patient consent statement

Authors confirm that a patient consent form has been obtained.
